# RhoA/ROCK1 regulates the mitochondrial dysfunction through Drp1 induced by *Porphyromonas gingivalis* in endothelial cells

**DOI:** 10.1111/jcmm.17796

**Published:** 2023-06-06

**Authors:** Qin Dong, Yuxiao Luo, Yuqing Yin, Yiwei Ma, Yingyi Yu, Liu Wang, Huishun Yang, Yaping Pan, Dongmei Zhang

**Affiliations:** ^1^ Department of Periodontics, School of Stomatology China Medical University Shenyang China; ^2^ Department of Periodontics and Oral Biology, School and Hospital of Stomatology China Medical University, Liaoning Provincial Key Laboratory of Oral Disease Shenyang China

**Keywords:** atherosclerosis, Drp1, endothelial cells, mitochondrial dysfunction, *Porphyromonas gingivalis*, RhoA/ROCK1

## Abstract

*Porphyromonas gingivalis* (*P. gingivalis*) is a pivotal pathogen of periodontitis. Our previous studies have confirmed that mitochondrial dysfunction in the endothelial cells caused by *P. gingivalis* was dependent on Drp1, which may be the mechanism of *P. gingivalis* causing endothelial dysfunction. Nevertheless, the signalling pathway induced the mitochondrial dysfunction remains unclear. The purpose of this study was to investigate the role of the RhoA/ROCK1 pathway in regulating mitochondrial dysfunction caused by *P. gingivalis*. *P. gingivalis* was used to infect EA.hy926 cells (endothelial cells). The expression and activation of RhoA and ROCK1 were assessed by western blotting and pull‐down assay. The morphology of mitochondria was observed by mitochondrial staining and transmission electron microscopy. Mitochondrial function was measured by ATP content, mitochondrial DNA and mitochondrial permeability transition pore openness. The phosphorylation and translocation of Drp1 were evaluated using western blotting and immunofluorescence. The role of the RhoA/ROCK1 pathway in mitochondrial dysfunction was investigated using RhoA and ROCK1 inhibitors. The activation of RhoA/ROCK1 pathway and mitochondrial dysfunction were observed in *P. gingivalis*‐infected endothelial cells. Furthermore, RhoA or ROCK1 inhibitors partly prevented mitochondrial dysfunction caused by *P. gingivalis*. The increased phosphorylation and mitochondrial translocation of Drp1 induced by *P. gingivalis* were both blocked by RhoA and ROCK1 inhibitors. In conclusion, we demonstrate that the RhoA/ROCK1 pathway was involved in mitochondrial dysfunction caused by *P. gingivalis* by regulating the phosphorylation and mitochondrial translocation of Drp1. Our research illuminated a possible new mechanism by which *P. gingivalis* promotes endothelial dysfunction.

## 1 INTRODUCTION

The primary pathogen of periodontitis,^1^ *Porphyromonas gingivalis* (*P. gingivalis*), is also intimately linked to the progress of other chronic inflammatory diseases in the body, including atherosclerosis,^2^ rheumatoid arthritis^3^ and Alzheimer's disease^4^. In human clinical trials and mouse models, *P. gingivalis* is often detected in atherosclerotic plaques.^5^, ^6^ Generally, *P. gingivalis* has been identified as an independent risk factor for atherosclerosis in some studies.^7^, ^8^, ^9^

Mitochondria are highly dynamic organelles that continuously perform coordinated fusion and fission movements. Alterations in mitochondrial structure and functions will result from the imbalance between fission and fusion. The previous study of our group found that *P. gingivalis* infection leads to an increase in endothelial mitochondrial fission. In addition, it is determined that Drp1 mediates *P. gingivalis*‐induced mitochondrial dysfunction, but the specific mechanism is unclear.^10^

Mitochondrial dysfunction is currently recognized as an essential factor of atherosclerosis.^11^, ^12^ Lu et al. found that platelet‐derived growth factor type BB can induce vascular smooth muscle cells (VSMCs) phenotypic switching, proliferation, migration and neointima formation by activating the ROS/NFκB/mTOR/P70S6K signalling pathway, which is one of the pathological processes of atherosclerosis.^13^ Yu et al. found mitochondrial DNA damage accelerates the progression of atherosclerosis using human aortic specimens and mouse models of atherosclerosis.^14^

Rho family proteins are small G proteins with GTPases, which are widely presented in eukaryotic tissues. RhoA (Ras homologous gene family member A) is one of the most critical Rho family members. RhoA serves as a molecular switch that cyclically regulates intracellular signalling between an inactive GDP binding conformation and an active GTP binding conformation.^15^ Rho‐kinase 1 (Rho‐related coiled‐coil containing protein kinase, ROCK1) is the direct downstream and primary effector substrate of RhoA.^16^ Phosphorylation of myosin phosphatase targeting subunit 1 (MYPT1), one of the important physiological substrates of ROCK1, facilitates interaction and phosphorylation of the catalytic domain of ROCK1.^17^ The RhoA/ROCK signal mediates the process of cardiovascular diseases by regulating biological processes such as inflammation, differentiation and apoptosis. In addition, some research suggested the RhoA/ROCK1 pathway regulates mitochondrial fragmentation through Drp1, a large GTPase,^18^ which is activated and transported to the surface of mitochondria to regulate mitochondrial fission. Shen et al. found that RhoA/ROCK1 pathway was engaged in phosphorylated Drp1 at the 616th Serine in cardiomyocytes pretreated with TNF‐α, which promoted mitochondrial fragmentation.^19^ Another report showed that in LPS‐pretreated mice, ROCK1 inhibitor could improve mitochondrial function by restricting excessive mitochondrial fission through inhibiting Drp1(Ser616) phosphorylation.^20^

Although we have learned that mitochondrial dysfunction caused by *P. gingivalis* infection depended on Drp1. However, the signalling pathway that regulates mitochondrial fragmentation and dysfunction in *P. gingivalis*‐induced endothelial cells remains elusive. Here, the role of RhoA/ROCK1 pathway and the further involvement of *P. gingivalis* in mitochondrial dysfunction were explored. Our findings would provide new clues to understand how *P. gingivalis* facilitated the formation of atherosclerotic lesions.

## 2 MATERIALS AND METHODS

### 2.1 Cell culture

EAhy926, a representative human umbilical vein endothelial cell line, was obtained from CellCook Biotech Company. It was grown under conditions of 37°C and 5% CO_2_ in Dulbecco Modified Eagle's Medium (Gibco BRL) supplemented with 15% foetal bovine serum. Our studies were carried out on cells in passages 4 to 6.

CCG‐1423 (APExBIO) selectively inhibits SRF‐mediated transcription of Rho signalling pathway activation.^21^ Y‐27632 (AbMole Bioscience) is a pharmacologically specific inhibitor of ROCK.^22^ They were used to determine the regulatory role of RhoA/ROCK1 pathway in *P. gingivalis* infection cells. Control cells were those exposed to DMSO only.

### 2.2 Bacterial culture

*Porphyromonas gingivalis* ATCC 33277 was inoculated in brain heart infusion broth containing 5% defibrillated sheep's blood, 0.1% vitamin K1 and 0.5% hemin. The bacteria were grown in anaerobic environments with 80% N_2_, 10% O_2_ and 10% H_2_. The cells were treated with *P. gingivalis* at different time points with a multiplicity of infection (MOI) of 100 in the following experiments. Cells that grew under the same conditions without infection were considered as a control.

### 2.3 Determination and quantification of the opening of mPTP by fluorescence staining and flow cytometry

The openness of mPTP in EA.hy926 cells was tested using the Mitochondrial Permeability Transition Pore Detection Kit (Beyotime). CoCl_2_'s capacity to quench the fluorescence of calcein in the cytoplasm served as the basis for the evaluation. CoCl_2_ can enter the mitochondria of open mPTP and combine with Calcein AM to cause partial or complete quenching of green fluorescence. Briefly, Calcein AM and CoCl_2_ were combined into the detecting buffer and then incubated with EA.hy926 for 30 min. The nuclei were stained with DAPI (Beyotime) before being visualized with the CLSM. The loss of green fluorescence indicates an increase in mPTP opening. The cells were stained, as mentioned above. Flow cytometry (FACS, Becton‐Dickinson) was used to gather fluorescence intensity, which was then analysed using FlowJo 10 analytic software.

### 2.4 Measurement of mtDNA copy number

TaKaRa MiniBEST Universal Genomic DNA Extraction Kits (Takara) were exploited for extracting the DNA of EA.hy926 cells. The mtDNA copy number was detected by real‐time PCR (RT‐PCR) using SYBR Premix Ex Taq II (Takara). With GAPDH serving as an internal reference, the relative copy number of mtDNA was determined by the 2^‐ΔΔCt^ method. The primers applied in RT‐PCR were as follows: mtDNA forward: 5′‐AACATACCCATGGCCAACCT‐3′, mtDNA reverse: 5′ AGCGAAGGGTTGTAGTAGCCC‐3′, GAPDH forward: 5′ CAGGAGGCATTGCTGATGAT‐3′, GAPDH reverse: 5′ GAAGGCTGGGGCTCATTT‐3′.

### 2.5 Determination of ATP contents

ATP Assay Kits (Beyotime) were utilized to ascertain the ATP production in the whole lysate of EA.hy926 cells. Cellular ATP levels of every group were computed according to the standard curves and then normalized to the control.

### 2.6 Western blotting

The protein concentration of cells was measured by a BCA assay. SDS‐polyacrylamide gel electrophoresis was used to isolate the same amount of protein, which was then transferred to a polyvinylidene fluoride membrane with GAPDH (1:3000; Affinity Biosciences) as an internal control. After 5% skim milk blocking, specific primary antibodies were used to detect the target proteins, including rabbit anti‐RhoA Antibody (1:1000; Bioworld Technology), rabbit anti‐ROCK1 Antibody (1:1000; BOSTER BIOLOGICAL TECHNOLOGY), rabbit anti‐MYPT1 Antibody (1:1000; BOSTER BIOLOGICAL TECHNOLOGY), rabbit anti‐p‐MYPT1 Thr696 Antibody (1:500; Bioworld Technology) and rabbit anti‐p‐Drp1 Ser616 Antibody (1:500; ABclonal). The blots were washed after overnight incubation and incubated for 1 h with goat anti‐rabbit IgG (1:5000; Abbkine, Inc.). The results were analysed by Odyssey CLX (LI‐COR) and ImageJ 1.8.0 software.

### 2.7 RhoA activity assay

The RhoA activation kit (STA‐403A; Cell Biolabs) was employed to value whether RhoA was activated. The GTP‐bound form of RhoA was pulled down by incubating the equivalent amount of protein and a predetermined amount of GST‐rhotekin‐RBD on a rotator at 4°C for 1 h. The beads were then centrifuged, washed, resuspended in 20 μL loading buffer and boiled. Western blotting was carried out to determine the pulled‐down GTP‐bound RhoA amount using an anti‐RhoA antibody.

### 2.8 Observation of mitochondrial morphology by transmission electron microscopy (TEM)

Cells were fixed using 2.5% glutaraldehyde for 24 h, followed by 1% osmium tetroxide for 2 h at room temperature. Subsequently, the sample was dehydrated, immersed, embedded, ultrathin sectioned and stained with lead citrate and uranyl acetate. Afterwards, the TEM (H7650; Hitachi) was used to observe the mitochondrial morphology.

### 2.9 Quantitative analyses of mitochondrial networking

The mitochondrial network refers to forming a highly interconnected network of tubular mitochondria. Cells were plated on the confocal petri dish for 24 h before being stained by MitoTracker Red (Solarbio) in a 37°C condition. Then, confocal laser scanning microscopes (CLSM; GeneTimes) were used to observe the mitochondrial network. Image‐Pro Plus 6.0 software was exploited to spatially process the obtained image using the ‘top hat’ filter to obtain a binary image that removed artefacts. Quantitative analyses of mitochondria were performed to obtain aspect ratio (AR: major axis/minor axis), shape factor value (FF: perimeter^2^/4π∙area) and mitochondrial length.^23^ A smaller value obtained indicated an increase in mitochondrial fragmentation, while a higher value represented that the shape of the mitochondria had become longer and more complex.

### 2.10 Immunofluorescence

MitoTracker Red CMXRos (200 nM, Solarbio) were utilized to stain the mitochondria of EA.hy926 cells for 30 min after *P. gingivalis* treatment. After washing, fixing, permeabilizing and blocking, the cells were treated with the rabbit anti‐p‐Drp1 (Ser616) antibody (1:400, CST, MA, US) at 4°C overnight. Then, p‐Drp1 (Ser616) was detected using a secondary goat anti‐rabbit antibody (1:100, Solarbio). The CLSM was used to capture the images.

### 2.11 Statistical analysis

The mean ± SD was used to summarize the results of three separate tests. In SPSS 17.0 software, one‐way anova and SNK test of multiple group comparisons were employed for statistical analysis. *P*‐value < 0.05 indicated a statistically significant difference.

## 3 RESULTS

### 3.1 Mitochondrial dysfunction induced by *P. gingivalis*

**FIGURE 1:**
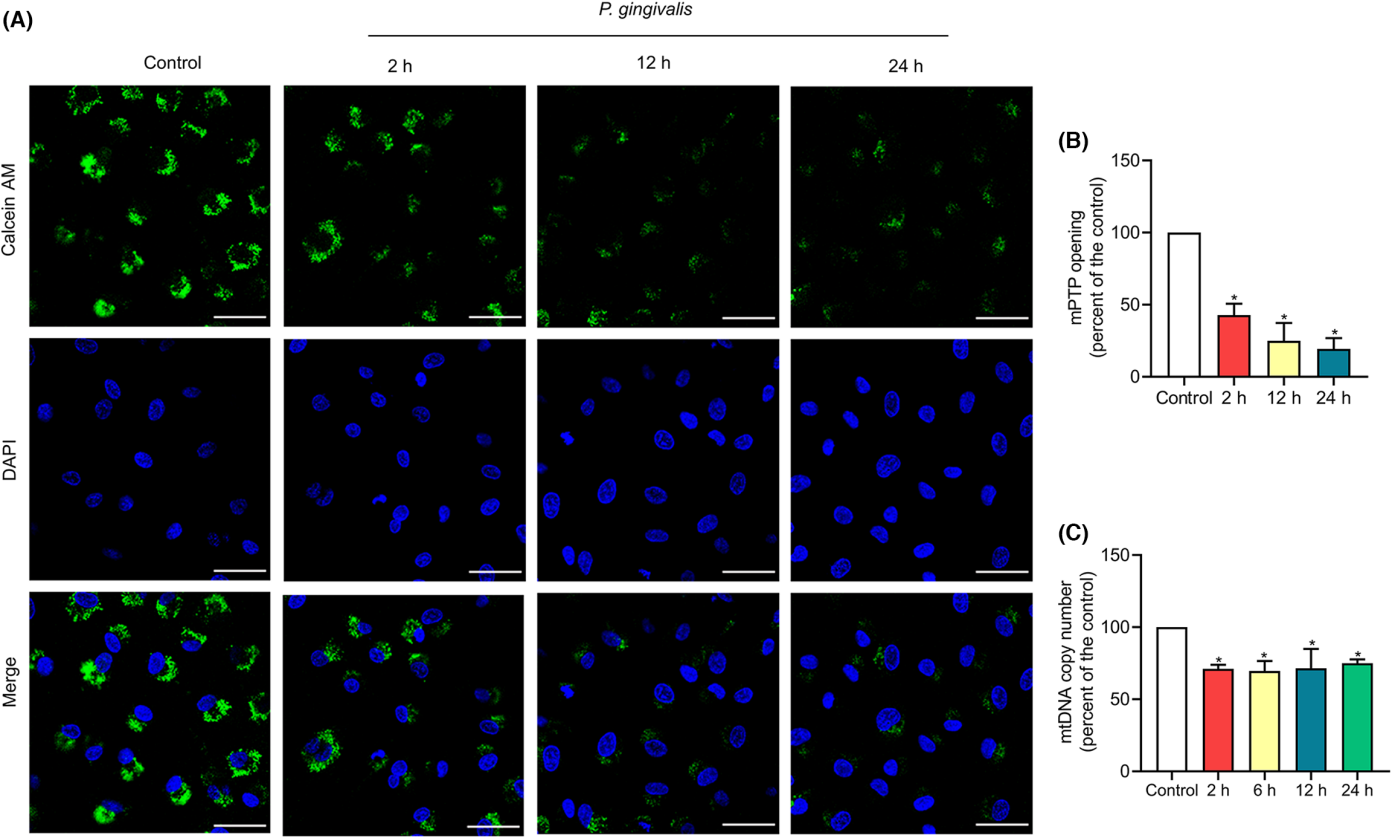
Effects of *Porphyromonas gingivalis* (*P*. *gingivalis*) on mitochondrial dysfunction. *P*. *gingivalis* was added into EA.hy926 cells at MOI = 100 for the times specified. Cells cultured without *P*. *gingivalis* were used as a control. (A) The opening degree of the mitochondrial permeability transition pore (mPTP) was determined by Calcein AM staining and confocal microscopy. Magnification 600; Scale bars: 50 μm. (B) Flow cytometry was used to quantify the openness of the mPTP. (C) Real‐time PCR was performed to determine the copy number of mitochondrial DNA (mtDNA). The data were presented as a percentage difference from the control group, which had been designed as 100%. The values represent the mean ± SD of three independent experiments.

According to our earlier research, *P. gingivalis* caused an accumulation in mtROS, a depolarization in mitochondrial membrane potential (MMP), and a drop in ATP levels.^10^ In the current exploration, we continued to investigate how *P. gingivalis* affects mPTP opening and mtDNA copy number by Calcein AM staining and RT‐PCR, respectively. The images showed that the fluorescence intensity of mPTP declined over time in infected cells (Figure 1A). Flow cytometry analysis corroborated these findings even more. As shown in Figure 1B, when mPTP fluorescence intensity was compared with controls, it was substantially reduced by 57.19%, 75.11% and 80.63% (*p* < 0.05) after 2, 12 and 24 h following *P. gingivalis* attack, respectively. Therefore, the conclusion in which *P. gingivalis* attack conspicuous induced mPTP opening was confirmed. RT‐PCR results showed that a significantly reduced mtDNA copy number was present in cells exposed to *P. gingivalis* (Figure 1C). Since cells were infected for 2 h, *P. gingivalis* had reduced mtDNA copy number, and the reduction was most significant at the 6‐h time point (30.27% reduction). After 12 and 24 h of infection, the mtDNA copy number rebounded slightly, and it was still lower than the control group, decreasing by 28.42% and 24.94% (*p* < 0.05), respectively.

### 3.2 RhoA activity and RhoA/ROCK1 pathway were activated by *P. gingivalis*

**FIGURE 2:**
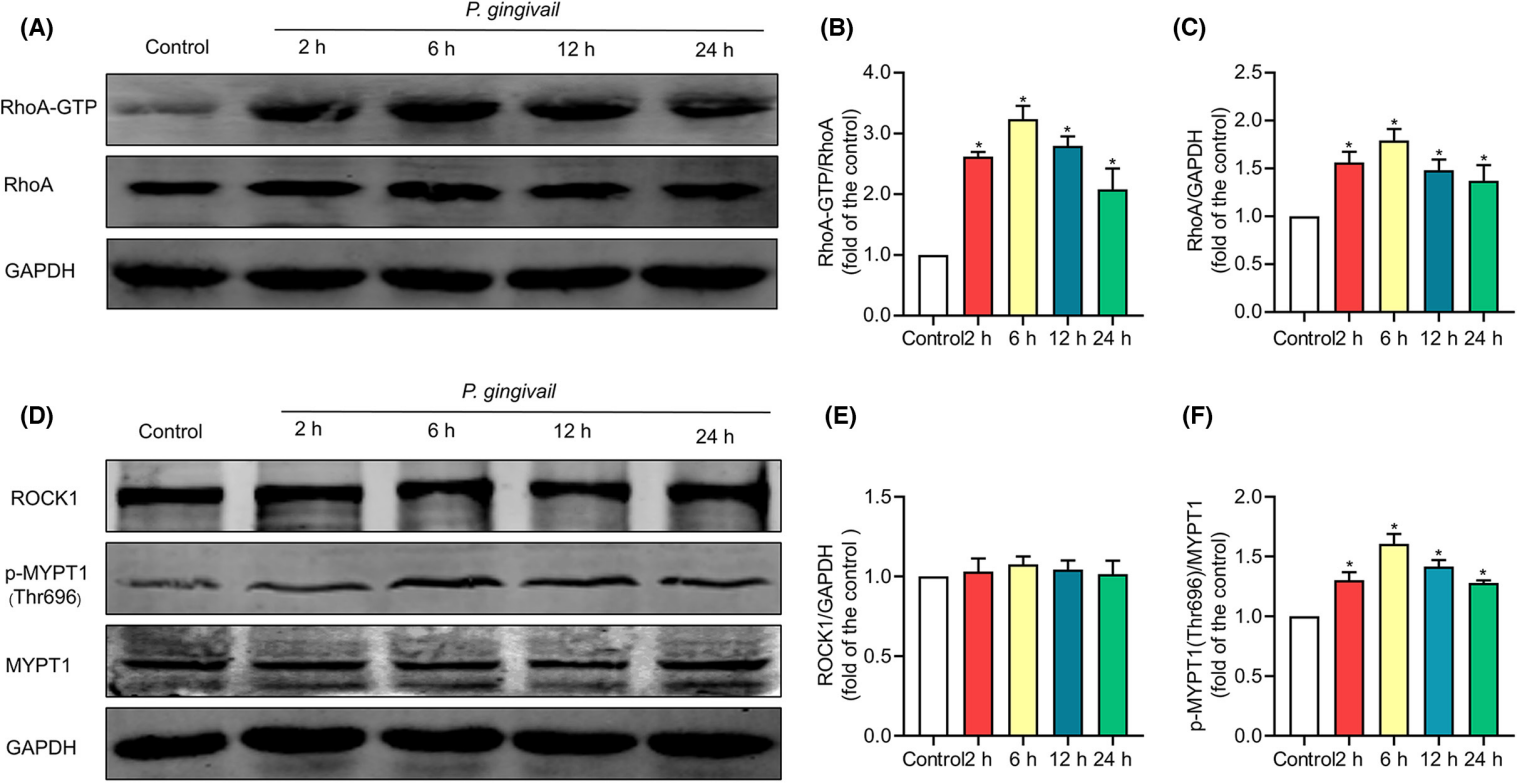
Effects of *Porphyromonas gingivalis* (*P*. *gingivalis*) on RhoA/ROCK pathway. *P*. *gingivalis* (MOI = 100) was applied to EA.hy926 cells for 2, 6, 12 and 24 h. Cells that had simply been exposed to a culture medium were used as controls. (A) The activation of RhoA was measured by a pull‐down assay. Western blotting was implemented to determine the protein levels of RhoA‐GTP and RhoA. (B) Quantitative analysis of the RhoA‐GTP in western blotting. (C) Quantitative analysis of RhoA in western blotting. (D) Western blotting was enforced to gauge the protein levels of ROCK1, p‐MYPT1 (Thr696) and MYPT1. (E) Quantitative analysis of the ROCK1 in western blotting. (F) Quantitative analysis of the p‐MYPT1 (Thr696) and MYPT1. The histogram showed the ratio of p‐MYPT1 (Thr696) to MYPT1. GAPDH was utilized as a loading control, and protein levels were quantified by band intensity. Mean expressions of the protein in all treated cells were contrasted with the control group, which was given an arbitrary value of 1. Data were given as the mean ± SD of three separate experiments. *p < 0.05 vs the control.

EA.hy926 cells that express RhoA and ROCK1 were employed, which was indicated by western blotting, to examine the impact of *P. gingivalis* on the RhoA/ROCK1 pathway. The pull‐down assay was utilized to evaluate the activation of RhoA following *P. gingivalis* exposure. It was revealed that the levels of RhoA and RhoA‐GTP increased significantly and reached a peak 6 h following *P. gingivalis* challenge (Figure 2A). The protein levels of RhoA in the infected cells increased by 1.79‐fold, and RhoA‐GTP increased by 3.23‐fold (Figure 2B, C) compared with the control. In contrast, *P. gingivalis* infection had no effect on ROCK1 expression (Figure 2D, E). We further determined the activation of ROCK1 by detecting the phosphorylation of MYPT1. As shown, *P. gingivalis* treatment significantly enhanced the phosphorylation of MYPT1 at Thr696 without affecting the total MYPT1 expression (Figure 2D). The *P. gingivalis* infection caused the level of p‐MYPT1 (Thr696) to reach a peak at 6 h, an increase of 0.61‐fold (Figure 2F) compared with the control cells. Collectively, the RhoA/ROCK pathway was activated by *P. gingivalis* infection.

### 3.3 The RhoA/ROCK pathway was engaged in *P. gingivalis*‐induced mitochondrial fragmentation

**FIGURE 3:**
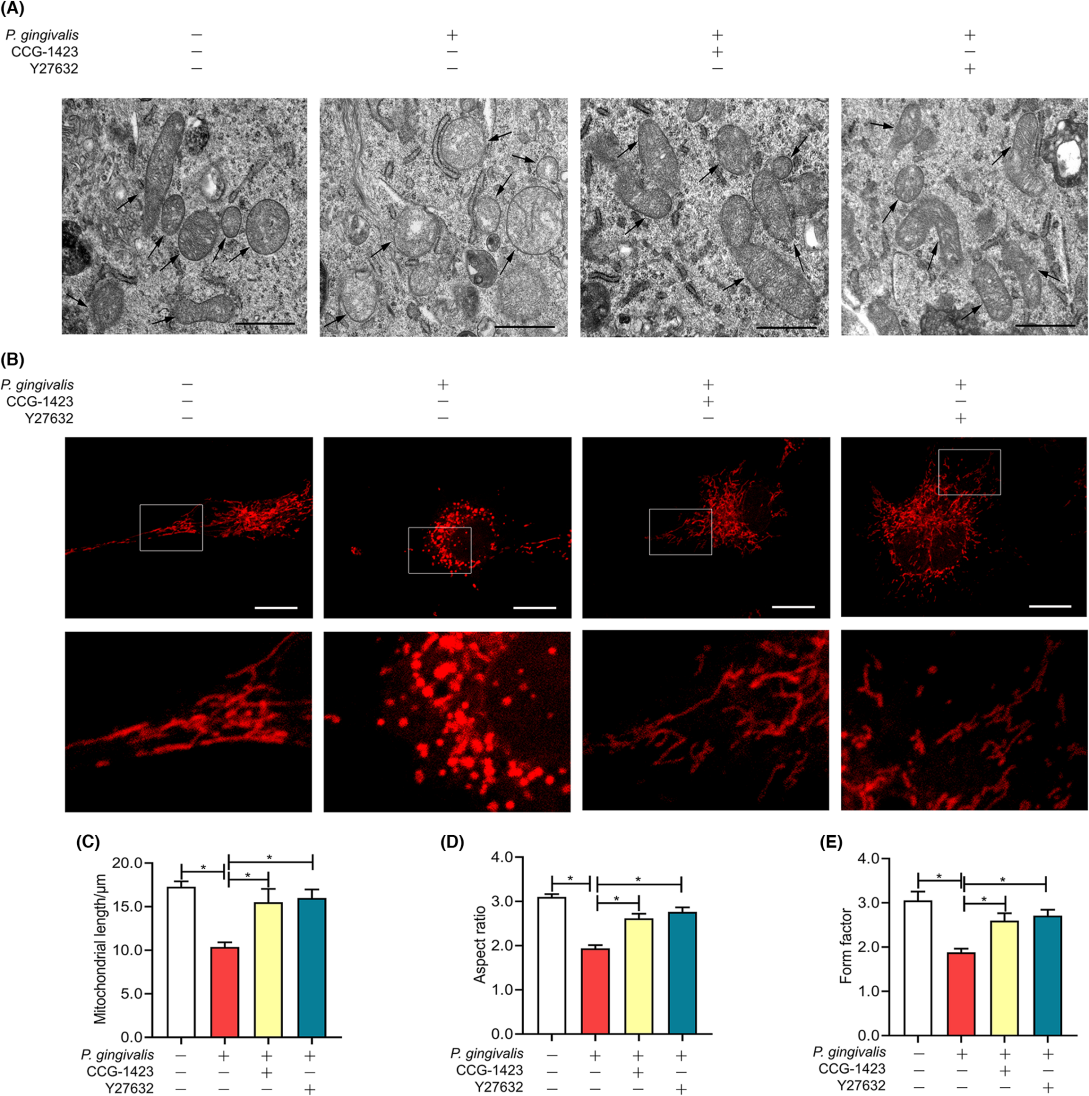
Effect of RhoA/ROCK pathway inhibition on *Porphyromonas gingivalis* (*P*. *gingivalis*)‐induced mitochondrial fragments. The cells were pretreated with DMSO, 10 μm CCG—1423 and 10 μm Y27632 for 30 min, respectively, and then exposed to *P*. *gingivalis* for 6 h. The cells were pretreated with DMSO only and then cultured in the medium were set as a control. (A) Transmission electron microscopy was used to observe the mitochondria morphology. Magnification 30,000; Scale bars: 1 μm. Arrowhead: mitochondria. (B) Before observing by a confocal laser scanning microscope, MitoTracker Red CMXR was operated to label the mitochondrial network. Magnification 2400; Scale bars: 20 μm. (C‐E) Summary data of B. Mitochondrial length, aspect ratio and form factor were calculated to estimate the mitochondrial size. Data were presented as the mean ± SD of three independent determinations. *p < 0.05.

To survey and evaluate the regulatory function of the RhoA/ROCK1 signalling in mitochondrial morphology, cells were observed by TEM. We observed that pretreatment with CCG‐1423 or Y27632 could inhibit the endothelial mitochondrial swelling and vacuole‐like changes caused by *P. gingivalis*, and most of the mitochondria of the cells returned to normal and rod‐shaped (Figure 3A). Confocal imaging also indicated the inhibition of the fragmentation and punctate changes of mitochondria by pretreatment of CCG‐1423 or Y27632 (Figure 3B). *P. gingivalis* stimulation for 6 h, AR, FF values and mitochondrial length were lowered by 38.39%, 37.49% and 40.02%, respectively (*p* < 0.05; Figure 3C, D, E). The mitochondria were elongated and cross‐linked into a network in the inhibitory groups compared with the *P. gingivali*s‐infected cells. The AR, FF value and mitochondrial length of the CCG‐1423 group were 1.35‐, 1.38‐ and 1.50‐fold that of the infected cells, respectively. Moreover, AR, FF values and mitochondrial length of the Y27632 group increased by 41.84%, 44.17% and 54.19%, respectively (Figure 3C, D, E). RhoA/ROCK1 pathway was shown to be implicated in mitochondrial fragmentation mediated by *P. gingivalis*.

### 3.4 Mitochondrial dysfunction induced by *P. gingivalis* was dependent on RhoA/ROCK pathway

**FIGURE 4:**
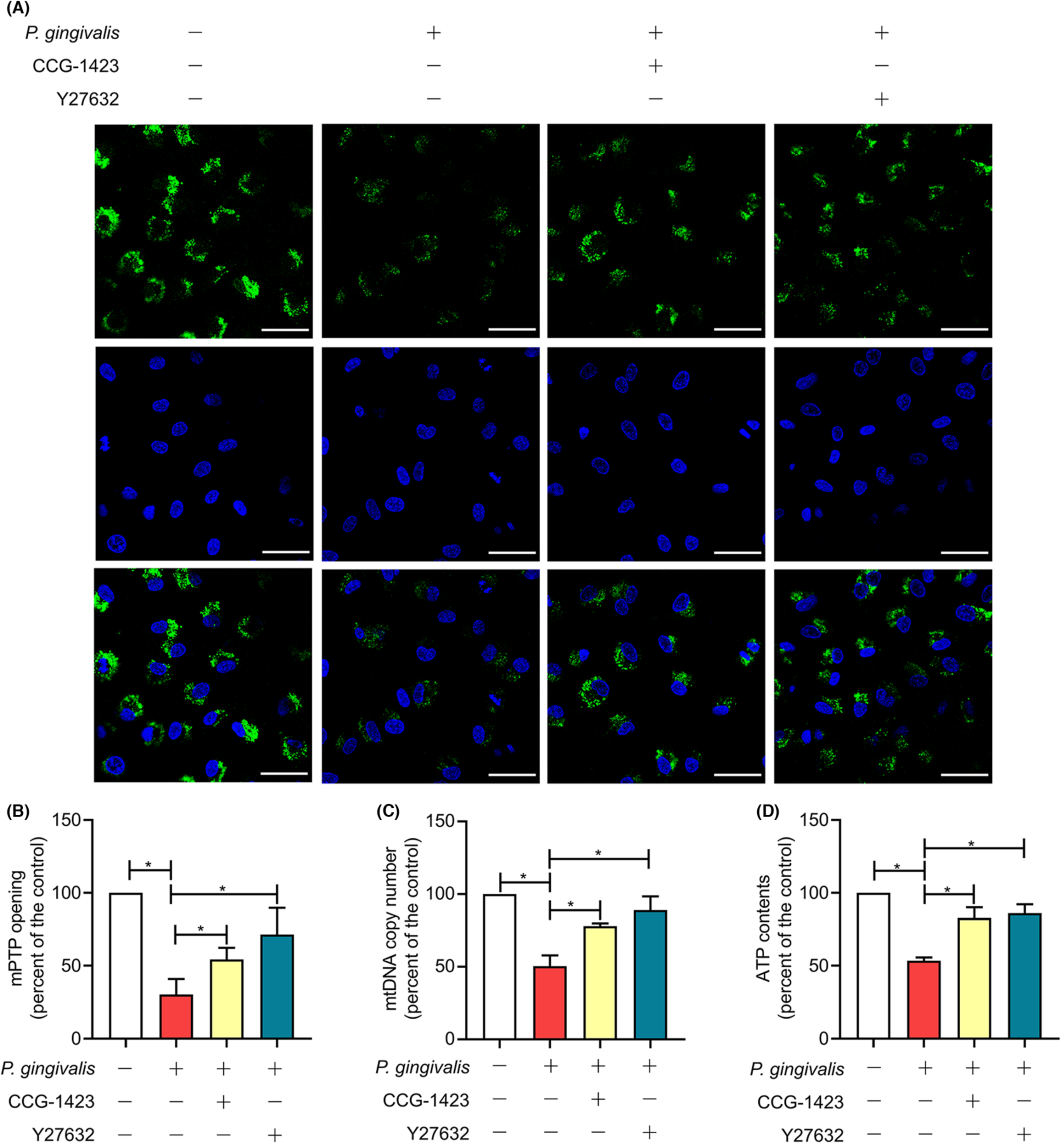
Effect of RhoA/ROCK pathway inhibition on mitochondrial dysfunction induced by *Porphyromonas gingivalis* (*P*. *gingivalis*). The cells were pretreated with DMSO, 10 μm CCG‐1423 and 10 μm Y27632, respectively, for 30 min before being infected by *P*. *gingivalis* (2 h for ATP, 24 h for mPTP and 6 h for mtDNA). The cells pretreated with DMSO only were considered as a control. (A) A confocal laser microscope was used to observe the openness of mPTP. Magnification 400; Scale bars: 50 μm. (B) The openness of mPTP was analysed quantitatively using flow cytometry. (C) mtDNA copy number was determined using real‐time PCR. (D) ATP contents were quantified. The data were represented as a change relative to the control group, which had been designed as 100%. Results were presented as the mean ± SD of three independent experiments. *p < 0.05.

The CLSM images in Figure 4A and B illustrated the impact of RhoA/ROCK1 pathway inhibition on mPTP. The green fluorescence intensity represents the openness of mPTP. In altering mitochondrial structure and function, mPTP is a vital participant. Stimulated by external factors, mPTP opening increases, mitochondrial membrane permeability increases and small molecules enter the mitochondrial matrix, resulting in mitochondrial swelling, calcium overload and reduced MMP and ATP production. Eventually, this leads to cell apoptosis or death.^27^ Flow cytometry analysis showed that after 24 h of infection, the fluorescence intensity decreased by 69.68% (*p* < 0.05) compared with the control, indicating that mPTP opening increased. However, this effect was inhibited by CCG‐1423 or Y27632 pretreatment. The fluorescence intensity of the RhoA and ROCK1 inhibitory groups rose considerably by 1.79‐ and 2.35‐fold, respectively, compared with the *P. gingivalis* group, which means that RhoA and ROCK1 inhibitors could attenuate the opening of mPTP induced by *P. gingivalis*. Consistent with the foregoing findings, the mtDNA copy number was pronouncedly reduced by 49.52% (*p* < 0.05) after 6 h of infection compared with control cells. Furthermore, the results showed that CCG‐1423 and Y27632 remarkably restored the reduction in mtDNA copy number induced with *P. gingivalis*. The mtDNA copy number increased by 54.49% in the CCG‐1423 group and 76.37% in the Y27632 group (Figure  4B, C, *p* < 0.05) compared with that in the infected group. Additionally, it was found that CCG‐1423 and Y27632 had similar effects on ATP contents. Compared with the control group, ATP contents were decreased by 46.62% (*p* < 0.05) 2 h after infection. However, in comparison with the infected group, pretreatment with CCG‐1423 and Y27632 increased the ATP contents by 55.17% and 61.22%, respectively, according to Figure  4C, D (*p* < 0.05). These results illustrated that RhoA and ROCK1 inhibitors effectively prevented the enhanced mitochondrial permeability, bioenergy deficiency and mitochondrial loss caused by *P. gingivalis*.

### 3.5 Phosphorylation of Drp1 (serine‐616) and mitochondrial translocation were induced by the activation of the RhoA /ROCK1 pathway

**FIGURE 5:**
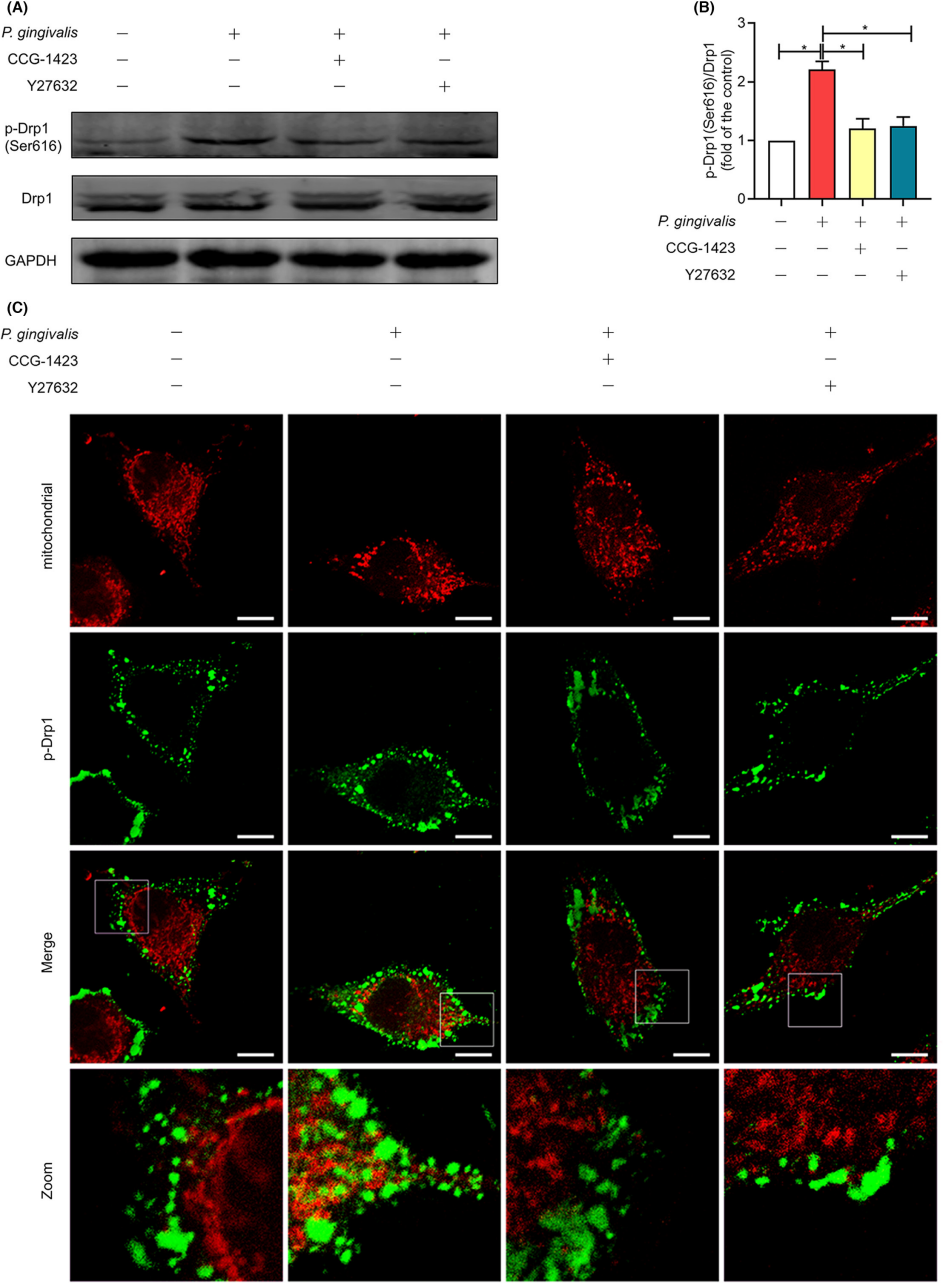
Inhibition of RhoA/ROCK pathway attenuated the expression and translocation of pDrp1(Ser616). Before 6‐h incubation with *Porphyromonas gingivalis*, the cells were pretreated with DMSO, 10 μm CCG‐—1423 and 10 μm Y27632 for 30 min, respectively. Control cells were only pretreated with DMSO. (A) Detection of Drp1 (Ser616) phosphorylation by western blotting. (B) The expression of p‐Drp1(Ser616) in western blotting was quantified. The relative protein levels were calculated in comparison with the controls, which were set 1. *p < 0.05. (C) The mitochondria and localization of p‐Drp1 (Ser616) were observed by a confocal microscope. p‐Drp1 (Ser616), green; mitochondria, red. The experiments were carried out three times to obtain representative pictures. Magnification 2400; Scale bars: 20 μm.

It has been demonstrated that *P. gingivalis* infection increases Drp1 phosphorylation and mitochondrial translocation in endothelial cells. The effect of the RhoA/ROCK1 pathway in the phosphorylation of Drp1 and mitochondrial translocation was investigated in this work. Compared with the control, *P. gingivalis* substantially upregulated the protein level of p‐Drp1 (Ser616) by 2.21‐fold. Besides, pretreatment with ROCK1 or RhoA inhibitors offset the induction of *P. gingivalis* (Figure 5B). In comparison with *P. gingivalis*‐infected cells, CCG‐1423 and Y27632 decreased p‐Drp1 (Ser616) protein levels by 45.28% and 43.35%, respectively (*p* < 0.05; Figure 5B). The location of p‐Drp1(Ser616) was assessed by further immunofluorescence analysis in the cell. Figure 5C demonstrates that the overlap of p‐Drp1 (Ser616) and mitochondria were considerably enhanced by *P. gingivalis*, while CCG‐1423 or Y27632 reduced p‐Drp1 (Ser616) foci on the mitochondria, according to those findings. In conclusion, the phosphorylation and mitochondrial translocation of Drp1 could be reduced by inhibiting the RhoA/ROCK pathway. These results illustrated that RhoA/ROCK1 regulated Drp1 phosphorylation and mitochondrial translocation.

## 4 DISCUSSION

Atherosclerosis is widely known to be the pathological basis of plenty of cardiovascular diseases. Multiple research has demonstrated an association between the activation of the RhoA/ROCK1 pathway and the onset and progression of atherosclerosis. It has been reported that ROCKs mRNA is enhanced in arteriosclerotic arterial lesions of animals and humans.^24^, ^25^ The elevated level of ROCK1 inhibits eNOS activity, an effector substrate of ROCK, and NO levels, resulting in impaired endothelial function and vasodilation, thereby accelerating the progression of atherosclerosis.^26^, ^27^ In addition, the RhoA/ROCK pathway plays a vital role in the formation of plaques and accelerates the process of atherosclerosis by mediating the differentiation of monocytes into macrophages and then secreting a series of inflammatory mediators.^28^ Generally, RhoA/ROCK1 pathway activation leads to the progression of cardiovascular diseases through inflammation,^29^ endothelial dysfunctions,^30^ VSMCs contraction,^31^ proliferation and migration.^28^ Nonetheless, the exact role of RhoA/ROCK in endothelial dysfunction induced by *P. gingivalis* is still unknown and needs further investigation.

Mitochondria serve as the cell's energy factory and are essential organelles for cell survival. They have received extensive attention from many scholars. Recent research has found that the imbalance of mitochondrial division and fusion leads to changes in mitochondrial dynamics, which is closely associated with atherosclerosis onset and progression.^32^ In the early stage of atherosclerosis, there will be changes in cellular inflammation, oxidative stress, endothelial dysfunction and VSMCs proliferation. Interestingly, mitochondrial dysfunction is thought to be related to these changes.^33^ The excessive production of ROS caused by mitochondrial dysfunction will oxidize cellular proteins, lipids and DNA.^34^ In the mouse model, the mitochondrial DNA damaged by the excessive accumulation of ROS will cause endothelial cell dysfunction and the proliferation of VSMCs, thereby accelerating the progression of atherosclerosis.^35^ It is also reported that the mtDNA damage of leukocytes is related to vulnerable plaques in coronary arteries. By assessing mtDNA in leukocytes and plaques in coronary patients, high‐risk plaques were associated with leukocyte mtDNA damage, and mtDNA damage was increased in atherosclerotic plaques than in normal arteries.^36^ As the RhoA/ROCK1 pathway is pivotal in atherosclerosis progression, and mitochondrial dysfunction is a recognized pathogenic mechanism of atherosclerosis, the relationship between the RhoA/ROCK1 pathway and mitochondrial dynamics has attracted our attention.

The effect of RhoA/ROCK1 on mitochondrial function has been extensively studied. Sebastien Preau et al. found that RhoA/ROCK activation leads to mitochondrial ultrastructural changes and reduced mitochondrial respiration in LPS‐treated mice.^20^ Hyun Tae Kang et al. showed that ROCK activation leads to the accumulation of mtROS and the decrease of MMP in Hutchinson–Gilford progeria syndrome fibroblasts. In contrast, ROCK inhibitor Y‐27632 can recover mitochondrial function, which is followed by the alleviation of the Hutchinson–Gilford progeria syndrome phenotype.^37^ Furthermore, it has been found that profilin‐1 (a small actin‐binding protein induced by advanced glycosylation end products) extensively dispersed in various cells leads to the accumulation of ROS, thereby activating the RhoA/ROCK1 pathway.^38^ These findings have shown that the activation RhoA/ROCK1 pathway is closely associated with mitochondrial dysfunction.

Recently, some academics have explored the specific mechanism of RhoA/ROCK1 pathway regulating mitochondrial dysfunction. Drp1, a GTPase, mediates mitochondrial fission by being transferred to mitochondria, which has been widely concerned.^39^ Its post‐translational modification, especially phosphorylation, can recruit its translocation to the mitochondria.^40^ Drp1 phosphorylation at the serine 616 site is regulated by multiple kinases, including AMPK,^41^ Ca^2+^/calmodulin‐dependent protein kinase,^42^ PTEN‐induced putative kinase 1,^43^ cyclin‐dependent kinase 1^44^ and ROCK.^19^ Among them, the RhoA‐activated signal is conducted to phosphorylation of Drp1 via ROCK1. Cameron S. Brand et al. reported that RhoA activation mediates mitochondrial fission through Drp1 phosphorylation and mitochondrial translocation in a ROCK‐dependent way in cardiomyocytes.^45^ According to a report by Ko, under high glucose conditions, ROCK1 activation in endothelial cells can phosphorylate Drp1 and lead to the production of mtROS and mtDNA damage, ultimately inducing mitochondrial autophagy and apoptosis.^46^ In addition, there are reports in the literature that the mitochondrial morphology and function are regulated by the RhoA/ROCK1/Drp1 pathway in human glomerular endothelial cells.^47^

Based on all the above viewpoints, it is supposed that the RhoA/ROCK1 pathway possibly regulates Drp1‐mediates mitochondrial dysfunction. However, whether the RhoA/ROCK1 pathway comes into play a role in endothelial mitochondrial dysfunction is unclear regarding periodontal infection. Growing evidence supports the opinion that *P. gingivalis* is defined as an independent risk factor for atherosclerosis.^48^ Its pili and LPS are involved in atherosclerosis formation by supporting the differentiation of monocytes into pro‐inflammatory macrophages and migration.^49^ Effective manipulation of adaptive immunosuppression through virulence factors is an essential mechanism of atherosclerosis associated with *P. gingivalis* infection.^50^ Here, *P. gingivalis* was used to observe the activation of RhoA/ROCK1 pathway and to explore the regulatory effect of RhoA/ROCK1 on Drp1, which has not been reported before.

The elevated expression and activation of RhoA by *P. gingivalis* were shown in this study. We observed ROCK1 activation through quantifying the expression of p‐MYPT1 (Thr696), although ROCK1 expression remained unchanged. As the downstream target of ROCK1, the phosphorylation level of MYPT1 increases, indicating ROCK1 activation.^51^ Our findings supported the opinion that the activation of RhoA/ROCK1 signalling was induced by *P. gingivalis* infection. Drp1 has previously been demonstrated to be a crucial protein for maintaining the balance of mitochondrial fission and fusion, essential for sustaining mitochondrial morphological characteristics and functions.^10^ Some other scholars have found that Drp1 is the direct substrate of ROCK1 and regulates mitochondrial fission. We hypothesized that RhoA/ROCK1 was the critical signalling pathway connecting *P. gingivalis* and mitochondrial fission. As predicted, inhibiting the RhoA/ROCK1 pathway downregulated the Drp1 phosphorylation and mitochondrial translocation, significantly alleviating mitochondrial fragmentation and dysfunction. This is in line with the RhoA/ROCK pathway's influence on fibroblast and glomerular endothelial cell mitochondrial fragmentation.^47^, ^52^

According to the report, the infection of bovine mammary epithelial cells with *Escherichia coli* increases mitochondrial fission mediated by Drp1, resulting in decreased MMP, the continuous opening of mPTP, and calcium ion disturbance.^53^ Jain et al. found that *Helicobacter pylori* can induce mitochondrial fragmentation in human gastric cancer cells and mouse embryonic fibroblasts through Drp1.^54^ Escoll et al. reported that *Legionella pneumophila* infection promotes the phosphorylation of Drp1 (Ser616) in macrophages, resulting in increased mitochondrial fission and decreased MMP, affecting cellular energy metabolism.^55^ Similarly, the current study found that infection of *P. gingivalis* resulted in the prolonged opening of mPTP and a decline in mtDNA copy number. The mPTP takes a pivotal part in the mitochondrial structure and functions. When mPTP continues to open in a stress environment, ROS are released, MMP decreases, mitochondrial swelling and calcium overload occur, and even pro‐apoptotic proteins that trigger cell death are released, leading to cell death.^27^ Mitochondrial function is closely related to the quantity and quality of mtDNA, namely the copy number and the integrity of mtDNA. The changes in copy number are regulated by oxidative stress, autophagy, replication and transcription‐related factors of mtDNA.^56^ mtDNA is more vulnerable to ROS attack when exposed to oxidative damage than nuclear DNA. Interestingly, a decrease of mtDNA copy number is proven to lead to endothelial cell dysfunction, which is a characteristic of early events occurring in the pathogenesis of atherosclerosis.^57^

Although we established an infection model with viable *P. gingivalis* and found that *P. gingivalis* induced mitochondrial dysfunction, we did not further confirm which virulence factor of *P. gingivalis* was responsible. It is well‐accepted that gingipains, LPS, peptidoglycan and flagellin are fundamental virulence factors for *P. gingivalis*. Among them, gingipains provide about 85% of the proteolytic activity and have been considered an essential virulence factor for *P. gingivalis*.^58^ Cao et al. treated myocardial cells with gingipains and observed disruption of mitochondrial integrity, inducing mitochondrial pathway apoptosis.^59^ *P. gingivalis* can degrade platelet endothelial cell adhesion molecule 1 and vascular endothelial cadherin through gingipains, leading to vascular injury, increased endothelial permeability and endothelial dysfunction.^60^ Here, we conjectured that *P. gingivalis* expanded the gap and enhanced the permeability of endothelial cells through gingipains, opening up a channel for the invasion of *P. gingivalis* and its toxic products. Subsequently, RhoA/ROCK1 signal pathway was activated, leading to mitochondrial dysfunction and endothelial damage. However, further research was required to confirm our assumption.

We have determined here that in *P. gingivalis*‐infected endothelial cells, RhoA/ROCK1 had a new role in the mitochondrial morphology and function depending on Drp1. The findings indicated a potential new mechanism that *P. gingivalis* promoted the occurrence and progression of atherosclerosis. Drugs that could inhibit the RhoA/ROCK1 pathway were expected to become new targets for treating atherosclerosis with *P. gingivalis* infection. To verify the viewpoints, however, further research is required.

## 5 CONCLUSION

Our findings revealed that RhoA/ROCK1 pathway was activated by *P. gingivalis,* and it was involved in the mitochondrial dysfunction depending on phosphorylation and mitochondrial translocation of Drp1 in the endothelial cells. Considering mitochondrial dysfunction plays a vital role in atherosclerosis, our current study might supply a new insight into the therapeutic target for atherosclerosis associated with *P. gingivalis* infection.

## AUTHOR CONTRIBUTIONS

**Qin Dong:** Investigation (lead); writing – original draft (lead). **Yuxiao Luo:** Data curation (equal); investigation (equal). **Yuqing Yin:** Data curation (equal); investigation (equal). **Yiwei Ma:** Data curation (equal); investigation (equal). **Yingyi Yu:** Data curation (equal). **Liu Wang:** Data curation (equal). **Huishun Yang:** Data curation (equal). **Yaping Pan:** Writing – review and editing (equal). **Dongmei Zhang:** Supervision (equal); writing – review and editing (equal).

## CONFLICT OF INTEREST STATEMENT

The author declares no conflict of interest.

## Data Availability

On reasonable request, the corresponding author could provide the data that support the finding of this article.
